# Report of the Committee Appointed to Examine into the Condition of the Mucous Membrane of the Intestinal Canal in Persons Dying of Cholera

**Published:** 1850-03

**Authors:** Samuel Jackson, John Neill, Henry H. Smith, William Pepper


*Report of the Committee appointed to examine into the condition of the mucous membrane of the intestinal canal in persons dying of Cholera.*

[The following "Report" made to the College of Physicians of Philadelphia, is copied from the printed "Transactions" of that body. The inaccessibleness of the original to the profession generally, and the value of the paper itself, will, it is hoped, be sufficient explanation for its appearance among the original communications. The gentlemen who compose the committee are eminently reliable, as regards accuracy and experience, and we regard the "Report" as one of the most valuable contributions to the pathological anatomy of cholera. It will be seen that they have refrained from expressing any opinion as deduced from these appearances, but have preferred leaving each one to make for himself the application of them in the investigation of the pathology of this disease.—Ed. Ex.]

Science is positive only when its facts are positive. A subject, the phenomena of which are numerous and complex, can be understood only when each of its phenomena or facts have been analysed, and positively ascertained.

In Epidemic Cholera, the most prominent and constant phenomena, are purging and vomiting: and in ninety or more, of one hundred cases, these phenomena appear to induce the condition that usually terminates fatally.

It is, therefore, an important object in determining the phenomena of Cholera, to ascertain whether any, and if any, what constant anatomical alterations can be detected in the intestinal canal of cholera patients who have succumbed under the disease.

The College, with the view to obtain, as far as possible, accurate information on this single question, appointed the undersigned, at the meeting held on the 19th day of June last, a committee to investigate this subject.

The Committee having attended to this duty, submit the following report:

The ordinary autoptical examinations, heretofore practised, have failed to yield any satisfactory information, and are nearly useless for the purposes of science.

Extensive structural lesions may exist, that cannot be seen, or very imperfectly discerned by the unaided sight, and without proper preparation.

It was determined by the Committee that the intestines, before being submitted to examination, should be finely injected, and subsequently inspected with the microscope.

This task was undertaken for the committee, by Dr. John Neill, demonstrator of Anatomy in the University of Pennsylvania. The admirable manner in which he has performed this duty, can be judged of by the beautiful preparations now on the table, which he has presented to the College for its museum.

The injections are made with turpentine colored with vermillion. It was found by Dr. Neill, that when he employed size, it did not penetrate well, and numbers of capillaries were not filled; the same result occurred when Canada Balsam was used. It led, at first, to the supposition that the capillaries were destroyed by the disease.

The committee, confining themselves strictly to the single object for which they were appointed, report the following facts as the result of their investigation.

1st. in the recent subject, the peritoneal coat, like all the serous membranes, was in all, remarkably dry. The lubricating serosity is deficient in the serous membranes.

2nd. The epithelial layer of the intestinal mucous membrane, was, in all the specimens, either entirely removed, or was detached, adhering loosely as a pulpy layer, mixed with mucus, or an albuminoid substance.

3rd. *Peyerian Glands.* Peyer's Glands, were developed to a greater or less extent in all the cases examined.

4th. *Solitary Glands.* These were also developed, and contained, in the recent subject, a minute quantity of white substance. These enlarged solitary glands have the appearances designated by Serres and Nonat, as Psorenterie.

The villi covering the glands of Peyer, and the solitary glands, present the same appearances as in other parts of the same intestine.

5th. *Villi.* They are denuded of the epithelial covering, but are unchanged in other respects.

6th. *Capillary Vessels.* These are entire, and manifest no departure from their normal state. The appearances of the capillaries of a cholera intestine, are identical with those of the healthy mucous membrane when the epithelium has been removed. In the natural state, the epithelium, from its thickness, conceals the injected capillaries.

In no instance was a vesicular eruption observed. In some of the dry specimens, there is an appearance that might be mistaken for it, but it is an emphysematous state, resulting from commencing putrefaction.

The foregoing facts are derived from the examination of twenty-five subjects.

Samuel Jackson, M. D.
John Neill, M. D.
Henry H. Smith, M. D.
William Pepper, M. D.

